# Humoral Responses and Serological Assays in SARS-CoV-2 Infections

**DOI:** 10.3389/fimmu.2020.610688

**Published:** 2020-12-18

**Authors:** Yannick Galipeau, Matthew Greig, George Liu, Matt Driedger, Marc-André Langlois

**Affiliations:** ^1^ Department of Biochemistry, Microbiology and Immunology, University of Ottawa, Ottawa, ON, Canada; ^2^ The Ottawa Hospital, Ottawa, ON, Canada; ^3^ uOttawa Center for Infection, Immunity and Inflammation (CI3), Ottawa, ON, Canada

**Keywords:** SARS-CoV-2, coronavirus, COVID-19, serology, humoral immunity, serological assays, original antigenic sin

## Abstract

In December 2019, the novel betacoronavirus Severe Acute Respiratory Disease Coronavirus 2 (SARS-CoV-2) was first detected in Wuhan, China. SARS-CoV-2 has since become a pandemic virus resulting in hundreds of thousands of deaths and deep socioeconomic implications worldwide. In recent months, efforts have been directed towards detecting, tracking, and better understanding human humoral responses to SARS-CoV-2 infection. It has become critical to develop robust and reliable serological assays to characterize the abundance, neutralization efficiency, and duration of antibodies in virus-exposed individuals. Here we review the latest knowledge on humoral immune responses to SARS-CoV-2 infection, along with the benefits and limitations of currently available commercial and laboratory-based serological assays. We also highlight important serological considerations, such as antibody expression levels, stability and neutralization dynamics, as well as cross-reactivity and possible immunological back-boosting by seasonal coronaviruses. The ability to accurately detect, measure and characterize the various antibodies specific to SARS-CoV-2 is necessary for vaccine development, manage risk and exposure for healthcare and at-risk workers, and for monitoring reinfections with genetic variants and new strains of the virus. Having a thorough understanding of the benefits and cautions of standardized serological testing at a community level remains critically important in the design and implementation of future vaccination campaigns, epidemiological models of immunity, and public health measures that rely heavily on up-to-date knowledge of transmission dynamics.

## Introduction

In late 2019, a novel betacoronavirus with sustained human-to-human transmission emerged from China’s Hubei Province (1, 2). This new coronavirus was identified as Severe Acute Respiratory Syndrome Coronavirus 2 (SARS-CoV-2) and is currently responsible for the worldwide Coronavirus Disease 2019 (COVID-19) pandemic (3, 4). Currently, a large proportion of the global population remains in various forms of temporary confinement to limit the spread of this virus, leading to significant disruptions in international travel and local socioeconomic activities. Thus, there is a pressing need to better understand the nature and duration of immunity against SARS-CoV-2 infection since nearly all epidemiological models, future vaccination campaigns, and public health measures assume that SARS-CoV-2 convalescence imparts some degree of immunity (5–7). Based on previous serological studies of SARS-CoV (the agent responsible for the 2003 epidemic) and of the Middle East Respiratory Syndrome coronavirus (MERS), neutralizing antibodies are relatively short lived, detectable for approximately three years following infection (8–11). However, the duration of immunity to these specific CoVs is not known. But according to reinfections frequencies by seasonal coronaviruses (sCoVs), this immunity may only last a year (12). Given the global spread and prevalence of SARS-CoV-2, this lethal virus is expected to become endemic (13).

As the pandemic continues its course and convalescent individuals recover, there is an increasing demand to develop validated serological assays that assess the antibody-mediated immunity conferred by a SARS-CoV-2 infection. The utility of serological assays in COVID-19 is manifold. From an epidemiological perspective, a validated serological assay could be used to identify the proportion of individuals exposed to the virus in various populations, such that the evolving disease incidence can be closely monitored. Measuring population seroprevalence can also be used to evaluate the prevalence of asymptomatic transmission and risk factors for acquiring the disease, which remain key research priorities. Furthermore, reliable serological assays are required to determine whether antibody titers, and more importantly neutralizing antibody titers, correlate with sterilizing immunity to SARS-CoV-2. These immunological features could prove to be robust predictors of the efficacy of future vaccines candidates. At the patient level, serological testing can be used as an adjunct to the current PCR-based assays to improve diagnostic sensitivity. Lastly, serological testing will have profound clinical and epidemiological implications by determining the duration and magnitude of immunity conferred by SARS-CoV-2 infection, characterizing the risk of reinfection, and predicting whether a given vaccine will require further boosters (14, 15). Ultimately, accurate serological data will be crucial for understanding the epidemiological and clinical characteristics of COVID-19 that must be established to inform effective and ethical response strategies to the COVID-19 pandemic, especially as policymakers discuss future approaches to resume economic activities and re-open borders.

Serological tests commonly use blood, serum, plasma, or saliva to detect multiple isotypes of circulating antibodies generated by B lymphocytes. Various private, academic, and public health labs are currently developing platforms for SARS-CoV-2 serological testing, utilizing technologies such as classical immunoassays (mostly Enzyme-Linked Immunosorbent Assays; ELISA), chemiluminescent immunoassays (CLIA), flow cytometry-based methods, and various other approaches, all with varying degrees of automation ranging from manual to high-throughput systems (16–20). Furthermore, point-of-care (POC) lateral flow immunochromatographic assays (LFAs) are becoming increasingly popular for their ease of use and rapid detection capabilities (21, 22). Although all serological testing methods share a common function in detecting antibodies against SARS-CoV-2, major differences exist among tests depending on the viral antigens being targeted, the subclass of antibody being detected, and the overall accuracy and reliability.

The urgency to produce serological assays has led to a recent surge in protocols, testing devices, and literature, each with varying degrees of quality and reliability. Here we review current advances in knowledge regarding the antibody response towards SARS-CoV-2 infection. We then look at current commercial and laboratory-based serological assays for SARS-CoV-2 and discuss their strengths and limitations as they relate to cross-reactivity, sensitivity, and specificity. Lastly, we investigate which epidemiological characteristics of COVID-19 may be gleaned from existing serological data, and how these can be applied to public health policy domains such as vaccination, herd immunity modeling, and other public health interventions.

## Down to the Basics: Antibody Classes and Class Switching

**Figure 1:**
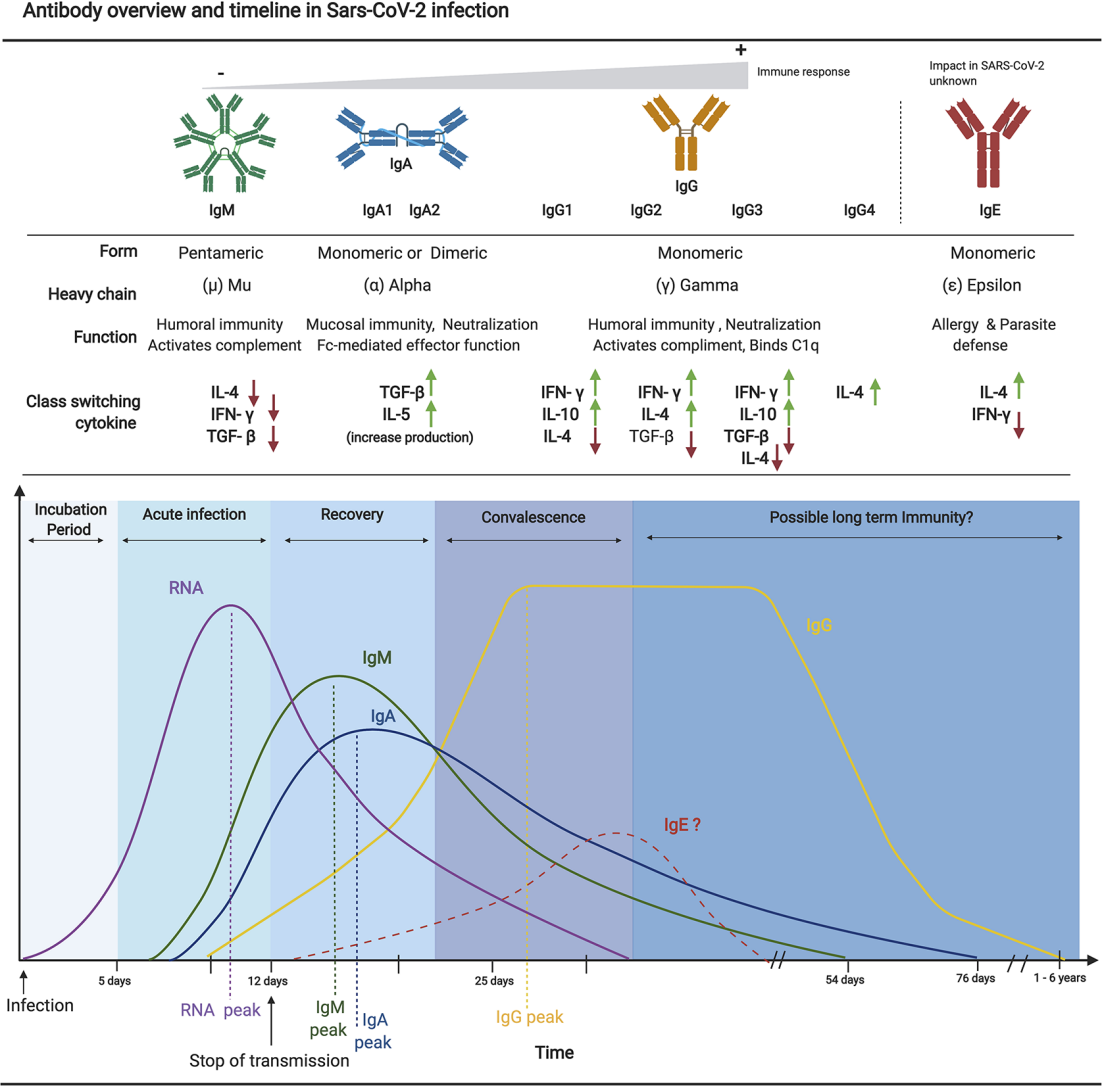
Overview of antibody isotype characteristics and an approximate timeline from SARS-CoV-2 infection to possible immunity. Each antibody isotype is represented with their typical form and associated heavy chain. A brief description of their main function as well as a representation of upregulated and downregulated cytokine necessary for each class switching is also included. The approximate timeline of appearance and subsequent decrease of each isotype in relation to the viral RNA is shown. The curves and values are based on recent serological studies discussed in this review. Since limited literature is available on the implication of IgE in the pathogenesis and antibody mediated immunity to SARS-CoV-2, as such the representation of the IgE timeline is purely hypothetical. Figures were generated using (23).

Multiple classes of antibodies (i.e., IgM, IgA, IgG, and IgE) are involved in antibody-mediated immune responses to viral infections (**Figure 1**). These classes are characterized by their intrinsic biophysical properties, functions, tissue distributions, and half-lives. Together with IgD, IgM immunoglobulins are normally the first to be expressed during naïve B cell development, comprising the majority of antibodies produced between B cell activation and class switching. IgM represents approximately 10% of all antibodies in the serum (24, 25). IgM antibodies demonstrate a relatively low affinity compared to IgG due to limited affinity maturation through somatic mutations. However, IgM antibodies demonstrate high avidity for the target antigen because they form pentamers that utilize multimeric interactions with the target antigen to facilitate neutralization (25). IgM antibodies are found mostly in circulation where they can facilitate antigen opsonization (26). Recent studies have also revealed diverse roles for secretory IgM in the mucosa of the gastrointestinal and respiratory tracts (27). Human IgA immunoglobulins, which can be further subdivided into the IgA1 and IgA2 subclasses (28), generally exceed levels of IgM in serum and are significantly more present in mucosal surfaces and secretions (i.e., saliva, breast milk, etc.) where they are central to mucosal immunity. IgA immunoglobulins form dimers upon secretion, which contributes to their increased avidity. Although IgA antibodies do not fix complement effectively like IgM, IgA antibodies secreted by plasma cells into the respiratory tract play a key role in mucosal immunity *via* pathogen neutralization, a process that facilitates aggregation and prevents the initial infection of host cells, thereby conferring sterilizing immunity to a pathogen (29, 30).

IgG antibodies start appearing later in the immune response because they undergo affinity maturation through somatic mutations, resulting in high affinity for the target antigen and a heightened capacity to neutralize pathogens (31). In addition to their role in neutralizing antigen, IgG antibodies also have other critically important roles, most notably Fc-mediated effector functions such as cell activations and antibody-dependent cellular cytotoxicity (ADCC) (32–34). IgG immunoglobulins are monomeric and represent about 75% of all antibodies in serum. They are associated with lasting immunity given their long half-life in blood and association with differentiated memory B cells (25). IgG can also bind C1q, activating the classical complement pathway of the innate immune system (35).

IgG antibodies can be subdivided into multiple subtypes (i.e., IgG1, IgG2, IgG3, and IgG4), each with slightly different roles in humoral immunity (32). For example, IgG1, IgG3, and occasionally IgG4 (upon repeated exposure) are secreted in response to protein antigens, while IgG2 almost solely responds to polysaccharide antigens (32). Given that different pathogens elicit different ratios of IgG subtypes, these can be used as characteristic profiles for monitoring the efficacy of vaccine designs with regards to correlates of protection (36, 37). Finally, IgE antibodies predominately mediate allergic reactions and immune responses against parasitic infections and comprise less than 0.01% of all total antibodies. IgE antibodies are monomeric and demonstrate a strong affinity for FcϵRI receptors expressed on numerous innate immune cells (e.g., mast cells, basophils, eosinophils), allowing for the generation of a generalized inflammatory response through innate immune system activation (38).

Current published data support that SARS-CoV-2 induces a classic viral response pattern, where IgM is the first isotype to appear, followed closely by IgA which peaks at 2-3 weeks post-symptom onset (PSO) before declining, and finally with IgG antibodies that remain detectable for several months PSO (39, 40). However, some studies have also reported the detection of virus-specific IgA responses preceding that of IgM, although the implications of this new pattern are not entirely understood (39, 41). Of particular interest, detectable levels of neutralizing antibodies against SARS-CoV-2 have been shown to start declining within three months of infection, especially among mild and asymptomatic cases (42–45). This, however, is not uncommon and resembles findings from patients infected with sCoVs (12, 46). Given the frequency of reinfections with sCoVs, this observation is likely a predictor of impermanent immunity and of heightened risk of reinfection in the short term.

## SARS-CoV-2 Viral Antigens

The effectiveness of an antibody response is largely dependent on the capacity of antibodies raised against native viral antigens during a natural infection, or against antigens in a vaccine, to act when exposed to the virus. These antibodies can either be present in blood, or produced *de novo* by memory B cells and plasma cells upon re-exposure to the viral antigens (47). Antibodies play a direct role in neutralizing incoming virus to prevent reinfections (i.e., sterilizing immunity), or by tagging viral antigens expressed on the surface of infected cells thereby triggering downstream Fc effector functions. In the case of CoVs, the viral antigens to which most antibodies are directed against are the viral spike (S) and nucleocapsid (N) proteins (48, 49).

**Figure 2:**
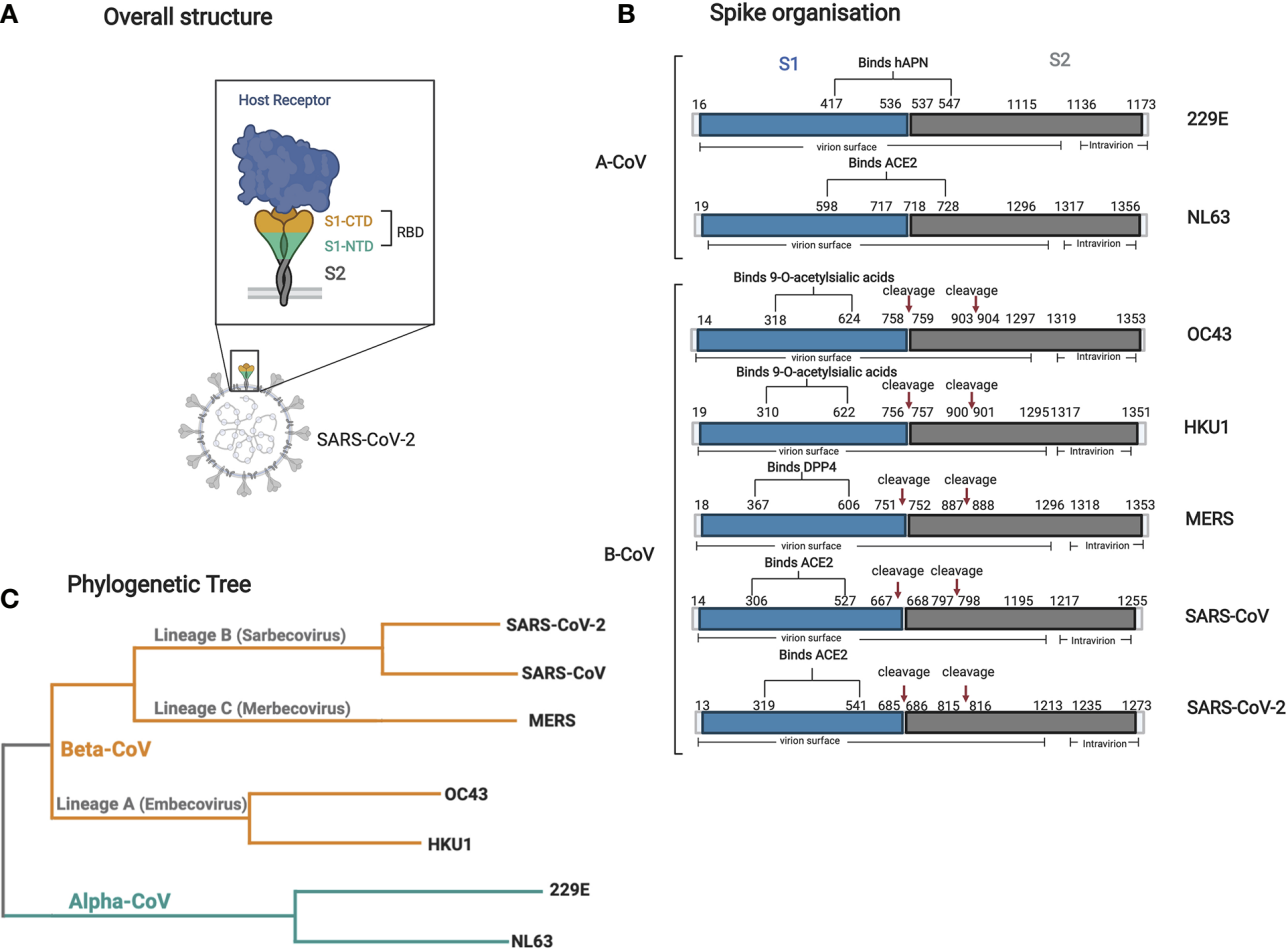
Structure and organization of the spike glycoprotein and phylogenetic tree of all seven human CoVs. **(A)** A cartoon structure of the Spike protein and its receptor (i.e., ACE2) is shown in relation to its localization on the virion surface. The S1 domain interacts directly with the receptor through its RBD *via* it’s C-terminal domain (CTD). **(B)** Graphical representation of the various spike human CoV proteins. The RBD, S1 (blue), and S2 (gray) domain locations and all other relevant sites (cleavage sites), and other topological features are shown with their respective amino acid sequence number. The information for each spike was obtained using Uniprot with the following accession numbers: 229E P15423, NL63 Q6Q1S2, HKU1 Q0ZME7, OC43 P36334, MERS K9N5Q8, SARS-CoV P59594, SARS-CoV-2 P0DTC2. **(C)** A phylogenetic tree based on the complete genome of all seven human CoVs was made using Clustal Omega multiple alignment tool using the reference genome sequenced from NCBI with the following accession numbers: 229E NC002645.1, NL63 NC005831.2, HKU1 NC006577.2, OC43 NC006213.1, MERS NC019843.3, SARS-CoV NC_004718.3, SARS-CoV-2 NC_045512.2. Figures were generated using (23).

The SARS-CoV-2 S protein is a trimeric transmembrane glycoprotein that is exposed on the surface of virions and mediates viral entry into host cells (**Figure 2A**) (50). This S protein constitutes the primary target of all current leading vaccine candidates (51). This large, exposed protein is readily targeted by neutralizing antibodies, which indirectly creates selective pressure for the emergence of evasion mutations. The propensity for S to mutate may limit its future use in serological assays and vaccines, as antibodies directed against the current variant may not bind emerging mutated epitopes, resulting in reduced vaccine efficacy while producing more false negatives in serological assays.

The S protein is further divided into two functional subunits, S1 and S2. S1 is responsible for binding to the host cell surface receptor ACE2 through its receptor-binding domain (RBD) found within subunit S1-, while S2 is involved in the fusion between the viral envelope and cellular membranes upon attachment (52) (**Figure 2B**). Along with orchestrating viral entry into host cells, the RBD region of S1 is of specific importance as many antibodies raised against RBD have neutralizing potential. Indeed, numerous viral epitopes that that are targeted by neutralizing antibodies are located within this region (53–57).

The viral N protein is an abundant nucleoprotein that binds the viral RNA genome and is contained within the virion. Each N protein contains three highly conserved and distinct regions: an N-terminal RNA-binding domain, a central Serine/Arginine-rich linker, and a C-terminal dimerization domain (58). The N protein has many functions associated with viral RNA packaging, RNA transcription, and viral replication. Since the N protein is abundantly expressed during infection, it is capable of inducing high levels of antibody production, making it a suitable target for serological assays (59–62). However, given that the N protein is not involved in viral entry and is shielded from antibodies by the viral envelope, most N protein antibodies are not likely to be neutralizing (63, 64). This was demonstrated by one study which showed that immunization with the SARS-CoV N protein induced antibodies with undetectable neutralizing activity (65).

## Testing for SARS-CoV-2 Antibodies

Serological tests are designed to detect the presence of antibodies against a given pathogen, in this case, SARS-CoV-2. A positive serological test result is indicative of a past exposure to one or several of the pathogen’s antigenic epitopes and therefore is not an indicator of an active infection. Furthermore, if the pathogen of interest shares antigenic epitope sequences with the proteins of other microbes or even that of vaccine antigens, a test can be reported as falsely positive. During a natural infection by SARS-CoV-2, the levels of viral RNA rapidly decrease during the second week and may become undetectable (66–68). Antibodies therefore become the primary and most accurate modality to detect a recently resolved or past infection (**Figure 1**). Serological tests are also critical for the detection of asymptomatic and previously undiagnosed infections in the population. This information is essential to guide public health interventions during an epidemic to mitigate the spread of a pathogen to the most vulnerable members of the population.

**Figure 3:**
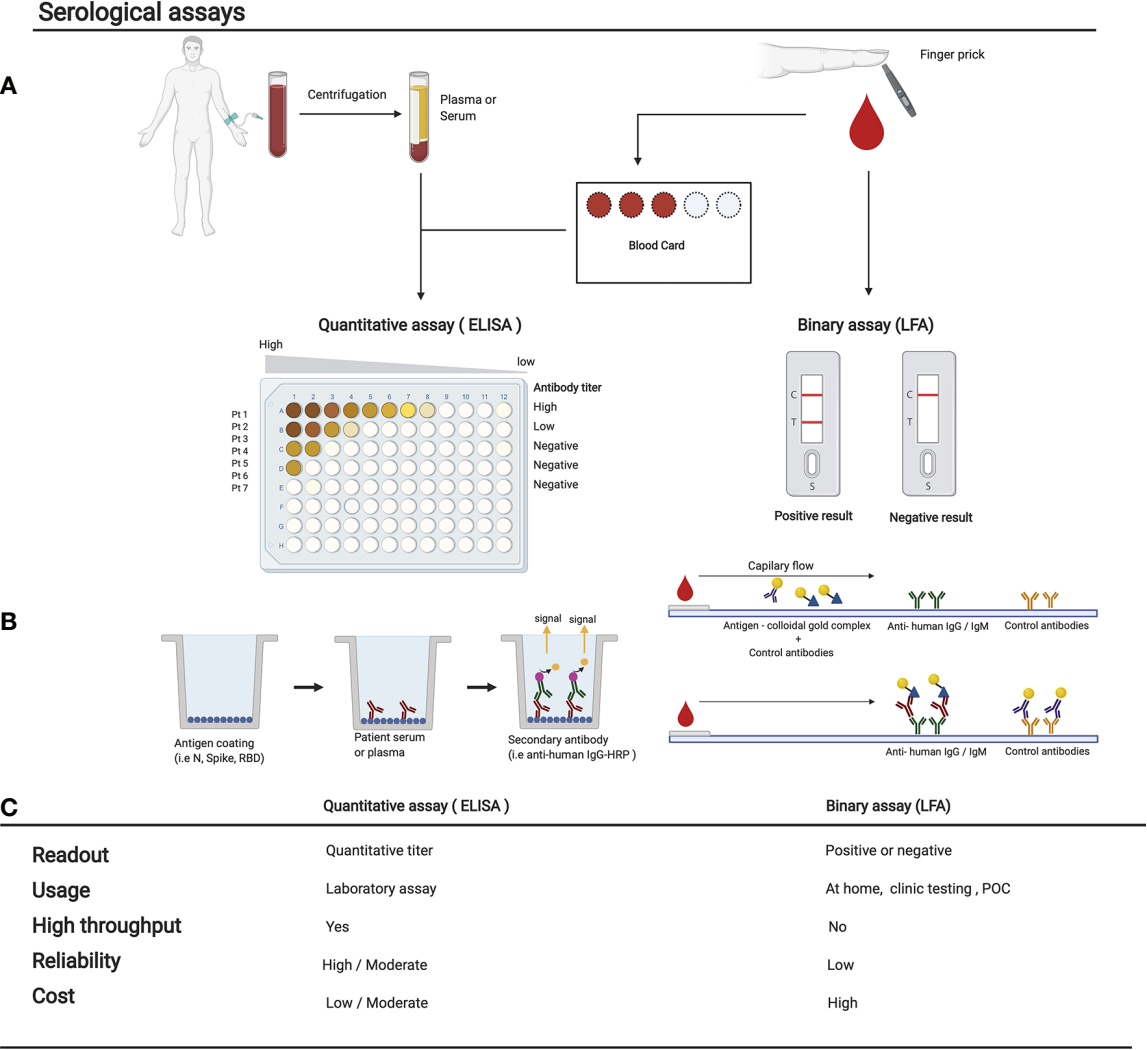
Comparison of various serological assays. **(A)** The sampling method and subsequent treatment of the blood before performing the serological assay is shown. Either a tube of blood is collected to isolate serum/plasma, or blood from a finger prick is used to fill a dried blood spot card or used directly in a LFA. Here we show the 2 main types of serological assays: on the left, a quantitative ELISA, or on the right, a binary result LFA. **(B)** The experimental procedure of each test is shown in their most simple form. Many variations are now available and are being used (see **Table S1**). **(C)** Comparison of the strengths and weaknesses of each method. POC, point of care; LFA, lateral flow assay. Figures were generated using (23).

Serological tests can be broadly divided into two categories: Rapid Diagnostic Tests (RDTs) and non-rapid tests (**Figure 3**) (49). A list of clinical serological tests currently approved are presented in **Table S1**. RDTs are most commonly LFAs which detect the presence of antibodies against multiple SARS-CoV-2 antigens within a 30-min time window. LFAs for SARS-CoV-2 detection work through the addition of a liquid sample (e.g., blood or saliva) – potentially containing the target antibodies – to one side of the testing device. The sample then diffuses by capillary action to a conjugation pad, where viral antigens conjugated to a colorimetric detection molecule (e.g., colloidal gold) are deposited. If SARS-CoV-2 antibodies are present, they will capture and dislodge these antigens and then migrate by continued capillary flow to a nitrocellulose membrane where anti-human capture antibodies are immobilized, usually anti-IgG and anti-IgM. If anti-SARS-CoV-2 antibodies are present, the colloidal gold (or another detection agent) will accumulate on a thin strip of anti-IgG and anti-IgM to create a colored line (**Figure 3**) (69). LFAs do not require multiple steps, nor the addition of any solution other than the patient sample. LFAs provide fast, qualitative, and easy-to-understand readouts that are designed for usage at home or in a POC setting without the need for equipment (70). Drawbacks of LFAs include their higher cost-per-test rate, their inability to analyze multiple samples simultaneously, their general lack of quantitative data, and importantly, a several-fold reduced sensitivity when compared to non-rapid testing methods (71, 72). Although RDTs are theoretically ideal for POC usage, recent studies have demonstrated that many newly developed RDTs for SARS-CoV-2 have failed to meet the necessary standards for sensitivity and specificity when compared to non-rapid testing (71–76). Therefore, for research purposes, LFAs are not the ideal choice.

Non-rapid serological testing methods include solid-phase immunoassays, microarrays, viral neutralizing tests, bead-based flow cytometry-based methods, and immunofluorescent microscopy, among others. These are all primarily laboratory tests that are carried out by trained personnel. Solid-phase immunoassays including ELISA, CLIA, Electrochemiluminescence Immunoassay (ECLIA), Enzyme-linked Fluorescent Assay (ELFA), and Dried Blood Spot ELISA (DBS-ELISA) are currently the most commonly used non-rapid, high-throughput methods for the detection of SARS-CoV-2 antibodies within a population (49).

Non-rapid tests generally involve the capture of primary SARS-CoV-2 antibodies within a saliva or blood sample to a solid support, like a dish or plate, coated with a SARS-CoV-2 antigen. This is followed by an initial wash step and the addition of a detection antibody, which is usually conjugated to a fluorophore or an enzyme like the horse radish peroxidase (HRP). Excess antibody is washed off and detection is performed. Colorimetric, fluorescent, and/or luminescent methods can be used as the final detection method depending on the detection antibody conjugate. Throughput can be easily scaled up using robotic liquid handlers.

Unlike LFAs, non-rapid tests can also provide valuable information on the quantity of each antibody within the samples (77). Non-rapid methods are generally much more sensitive than RDTs. Low-level antibody detection is especially important in the first 7 to 21 days of SARS-CoV-2 infection, when IgG levels are still rising, as well as >2 weeks post-infection when IgM antibodies begin to diminish (**Figure 1**) (67). Although most non-rapid tests currently use venous serum or plasma, or saliva samples in a liquid phase, DBS-ELISAs provide a practical alternative to venous samples by using only a few microliters of blood taken by pinprick and deposited onto an absorbent paper (**Figure 3**). Antibodies can then be eluted from a circular punch taken from the paper using a small amount of buffer. The ELISA is then performed in a standard fashion. This simple and convenient approach to collecting blood eliminates the need for a healthcare provider to perform venipuncture and provides an opportunity to conduct large-scale population-level seroprevalence studies using high throughput liquid handlers to perform DBS-ELISAs (78).

## Neutralization Assays and Their Importance

Although serological tests can determine prior immune exposure to SARS-CoV-2, neutralization assays provide critical knowledge on whether the detected antibodies are capable of neutralizing the virus and providing likely protection upon subsequent exposure. Currently, the most reliable neutralization assays involve live authentic SARS-CoV-2 viruses produced in cell culture and therefore require all procedures to be carried out in a Biosafety Level 3 (BSL3) facility (79). A current challenge is to develop a reliable neutralization assay that can be carried out in a standard BSL2 laboratory, at home, or in the clinic. To circumvent biosafety containment requirements, researchers are currently developing lab-based assays using viruses that consist of a less-harmful or non-infectious virus, such as murine leukemia virus (MLV) or vesicular stomatitis virus (VSV), pseudotyped with the SARS-CoV-2 S glycoprotein (80, 81). Other options include assays that utilize purified ACE2 to determine the effect of neutralizing antibodies on the ACE2-Spike interaction without the requirement of live cells or viruses. One example of a neutralization assay is the cPass SARS-CoV-2 Neutralization Antibody Detection Kit from GenScript Biotech. This test kit is advertised to test for pan-Ig neutralizing antibodies using the SARS-CoV-2 RBD as the viral antigen for antibody capture (82). Other promising surrogate neutralization assays have been proposed which utilize an ELISA-based competition binding assay against ACE2 (83, 84). Antibodies against RBD have been shown to be the primary source of neutralizing antibodies against the virus (53, 85–88). However, it should be noted that not all antibodies that bind RBD demonstrate neutralization potential, and that anti-RBD antibodies capable of neutralization may only be present at very low concentrations in some individuals post-infection (89, 90). Furthermore, RBD is not the only viral antigen that is a target for antibody-mediated neutralization; additional non-RBD epitopes elsewhere on the S protein have also been shown to neutralize the virus when targeted by antibodies (57, 91, 92). One caveat of the aforementioned neutralization assays is that they provide limited information on possible Fc-dependent effector functions, which likely also play an important role in protecting against SARS-CoV-2 (93). Therefore, current single-antigen neutralization assays only detect a subset of the total pool of neutralizing antibodies.

Of note, several commercial serology tests use the N antigen for antibody capture (**Table S1**). Given the high abundance of anti-N antibodies, targeting this viral antigen has the potential to increase test sensitivity (94). However, N proteins are located on the interior surface of intact viruses, and thus remain inaccessible to circulating antibodies. Therefore, tests that use N are unlikely to identify neutralizing antibodies that provide sterilizing immunity upon infection. Nevertheless, effector functions of anti-N antibodies could still provide protection (64). Currently, the RBD and S proteins are the most reliable antigens for measuring the abundance of neutralizing antibodies.

## Sensitivity and Specificity of Serological Assays for SARS-CoV-2

The quality and usefulness of a serological test is primarily evaluated by its degree of sensitivity and specificity (95). Sensitivity describes the ability of a serological test to provide a positive result from samples that contain antibodies against SARS-CoV-2 (“true positives”). Thus, a highly sensitive test would have a very low frequency of false negatives. Meanwhile, specificity describes the ability of a test to provide a negative result when a sample does not contain SARS-CoV-2 antibodies. Thus, a high specificity SARS-CoV-2 serological assay would have no or few false positives, including those resulting from cross-reactivity to any of the other six human CoVs (96, 97). The sensitivity and specificity of an assay are influenced by the cut-off point at which a test result is deemed positive. A receiver operating characteristic (ROC) curve is a useful graphical tool to visualize the relationship and trade-off between the sensitivity and specificity of an assay (98), but its details are beyond the scope of this review.

Serological tests that target IgM, which naturally has a lower affinity for the viral antigen than IgG, will be at a higher risk of producing false positives, and therefore should require a higher specificity threshold. Testing thresholds for specificity and sensitivity are arbitrary values established experimentally and differ between serological tests and methods. SARS-CoV-2 thresholds are primarily determined based on test results of negative control samples collected prior to the pandemic, as well as on positive control samples that have been confirmed by a certified clinical RT-PCR diagnostic test (99). Currently, there are no international reference standards for reporting test sensitivity and specificity, making it very challenging to compare the different serological tests and assays without carrying out a direct experimental comparison. Recent studies have sought to compare multiple testing kits with a small group of common samples (100–102). While this represents progress, what is ultimately required is a well-characterized set of standard sera that could be tested against any approved serology testing kit, allowing for the sensitivity and specificity of these kits to be compared (103). Other variables that require standardization for serological testing and kit comparisons include the length of time PSO for samples to be collected from patients, since the sensitivity and specificity of commercial tests can differ depending on the time at which the sample is collected (104), as well as the method by which samples are sometimes inactivated for lab safety (97, 105).

Sensitivity and specificity thresholds are also important for epidemiological considerations unique to certain situations and environments. These thresholds can be altered to allow for greater sensitivity of testing at the expense of specificity, or the opposite, whereby specificity is favored at the expense of sensitivity. For example, in a region with high SARS-CoV-2 seroprevalence, sensitivity may be prioritized over specificity to ensure the majority of positive cases are identified. The opposite is also true in low-prevalence regions (16). If the prevalence in a given region is very rare, then higher specificity and relatively lower sensitivity would be favored so that fewer patients would have false-positive results while still detecting the majority of true positives. It is essential to have the correct balance between sensitivity and specificity, as the epidemiological implications of disproportionate false negatives or false positives can be profound. A test with too many false positives will keep people isolated for longer than necessary, creating otherwise avoidable social and economic strains. A test with too many false negatives will result in the underestimation of disease prevalence, which may lead to a premature easing of disease containment policies and resurgent waves of infection as misidentified patients unknowingly continue to transmit the disease (16).

## Dynamics of the Antibody-Mediated Immune Response to SARS-CoV-2

Understanding the temporal profile by which circulating antibody classes are produced following SARS-CoV-2 infection is essential for the interpretation and clinical application of serological test results. During a viral infection, plasma B lymphocytes of the adaptive immune system produce different classes of antibodies in response to temporally regulated cytokine expression in a process called class switching or class switch recombination (CSR) (106). A given infecting pathogen type normally induces a characteristic cytokine profile that is responsible for triggering CSR for the production of the various isotypes and subtypes that are optimally suited to neutralize that type of microbe. Individuals suffering from severe COVID-19 are known to exhibit a dysregulation of pro-inflammatory cytokine release, also known as a cytokine storm (107–110). How this large release in cytokines alters the humoral response compared to asymptomatic, mild or moderate COVID-19 cases that do not exhibit this same cytokine storm is not yet clear.

While it is expected that IgM immunoglobulins are the first class detected following infection by SARS-CoV-2, as supported by a number of studies (37, 39, 40, 111), others have paradoxically demonstrated IgG responses that precede the IgM response (38, 112–115). This surprising discrepancy is likely related to cross-reactivity with pre-existing immunity to sCoVs (40, 111, 116–118). Nevertheless, the largest body of evidence suggests that nearly all SARS-CoV-2 infected individuals begin to produce IgM, IgA, and then IgG by 1 to 2 weeks PSO (**Figure 1**) (39, 90, 114, 119, 120). In fact, IgM antibodies against the viral N protein have been detected as early as 1 to 7 days PSO in 85% of individuals (39). However, these figures vary considerably depending on the type of serological assay and target antigen used. For example, *Long et al.* detected IgM antibodies against N protein in only 12% to 40% of cases during this same time period (i.e., 1–7 days) (121). The majority of patients appeared to have seroconverted by day 14 PSO, with approximately 94% of infected individuals having detectable levels of IgM against the RBD of the SARS-CoV-2 S protein, and 88% against the N protein (114, 122). IgM antibodies decline rapidly at approximately 20 days PSO, becoming undetectable at 60 days PSO on average (**Figure 1**) (123, 124). The impermanence of the IgM response suggests that the diagnostic role for IgM serology is most relevant in the detection of current and recent infections, within the first 1 to 2 weeks PSO, at which point its sensitivity for the diagnostic of an active infection may actually exceed that of PCR (39, 125, 126).

IgG immunoglobulins broadly have the most significant implications with respect to serological testing and antibody responses, given its high affinity for the antigen, capacity for viral neutralization, ability to activate complement, and predominant role in long-term immunity following infection or vaccination. Indeed, serological studies on other human CoVs including SARS-CoV and MERS have found the IgG antibody class to yield assays with greater specificity compared to IgM, to be significantly longer-lasting in comparison to IgM and IgA (127, 128), and to have strong links to neutralization and patient outcomes (114, 122). During acute SARS-CoV-2 infection, class switching from IgM to IgG occurs relatively quickly, with a median time to IgG detection ranging from as early as 7 days (120, 121) to approximately 14 days (39, 125, 129). IgG production also peaks later and is much slower to decrease than IgM (124, 130). The duration and intensity of the reported IgG antibody response for SARS-CoV-2 varies according to several study parameters that include disease severity and outcome, and antigens used in the serology assays. One study demonstrated an important reduction in IgG over 8 weeks in both symptomatic and asymptomatic cases, with many patients becoming seronegative during the study period (40% asymptomatic, 12.9% symptomatic) (121). Such observations are also supported by a number of additional studies that also measured a decline of IgG antibodies after several weeks PSO (44, 131). However, most groups have demonstrated that IgG levels against SARS-CoV-2 remained relatively stable within a 3 to 5 month observation period PSO (43, 124, 130–135).

Interestingly, among individuals infected by SARS-CoV-2, detectable antibody subtypes included RBD-specific IgG1 & IgG3, but rarely IgG2 or IgG4 (136). If a consistent subtype ratio is reliably established, this could help in identifying true seropositive individuals (convalescent from SARS-CoV-2) as opposed to false-positives with cross-reactive antibodies (convalescent from other sCoVs). Similar to IgM, IgA antibodies are produced shortly after PSO, with a median time to detection of 1 to 2 weeks PSO (39, 40, 67, 128). However, while IgM peaks at approximately 10 to 12 days, IgA levels appear to be relatively more persistent, peaking at approximately 20 to 30 days PSO (39, 123, 124, 133). To date, there is no serological evidence for the induction of IgE production in patients with COVID-19.

## Duration of SARS-CoV-2 Neutralizing Antibodies

Nearly all individuals who become infected with SARS-CoV-2 develop antibodies and neutralizing antibodies following infection, demonstrating a successful adaptive antibody-mediated immune response (43, 45, 87, 88, 110, 121, 123, 124, 130, 134, 137). This is consistent with non-human primate (NHP) studies where exposure to the live virus provided protection against reinfection without clinical illness, with corresponding neutralizing antibody responses (138, 139). Therefore, regardless of the discrepancies between studies regarding persistence or decline of total antibodies, it is imperative to note that persistence of IgG antibodies does not necessarily imply persistence of neutralizing antibodies during this same period. In fact, most studies report various intensities of decline in neutralizing antibodies after three months PSO, with disease severity being a factor strongly correlating with the decay rate of neutralization (42–44, 90, 121, 124, 140).

In contrast, data from some studies have indicated that neutralizing antibody titers remain stable ranging from 75 days to 6 months PSO in COVID-19 convalescent individuals with a broad spectrum of disease severity (130, 134, 141, 142). In particular, a large cohort study by Wajnberg et al. analyzed humoral responses in 30,032 antibody-positive individuals in New York City, and demonstrated relatively stable anti-S IgG antibody titers over five months, with these titers correlating with virus neutralization (130). Similar findings were demonstrated in a convalescent cohort study in China over a six month follow-up period with anti-S and anti-N IgG antibodies detectable in 70% of patients, with associated stability in neutralization titers, although these results are yet to be peer-reviewed (141).

One factor that could influence the persistence of neutralizing antibodies within specific cohorts is a high prevalence of the virus in a defined geographical region or in a specific subpopulation of individuals such as frontline healthcare workers. Regular re-exposure to the virus may help sustain higher antibody and neutralizing antibody levels. A second factor may be the persistence of antigens in tissues or as immune complexes on follicular dendritic cells. In fact, new evidence suggests that memory B cell responses continue to evolve in recovered individuals for at least six months after infection (143). During this time, somatic mutations accumulate to produce neutralizing antibodies with increased potency. This suggests that regardless of whether neutralizing antibodies wane over time, re-exposure to the virus is likely to stimulate memory B cells to mount a rapid and effective humoral response.

Taken together, while the specific conditions that influence the total duration of SARS-CoV-2 humoral immunity remain to be more precisely defined, decreasing antibody titers do not necessarily imply waning or defective immunity. In fact, antibody titers are expected to decrease following the resolution of an acute infection as a natural consequence of the depletion of short-lived plasma cells when immediate and sustained immune responses are no longer necessary (144, 145). Furthermore, the half-life of IgG in serum is about 26 days (146). Without continuous antibody output from plasma cells, antigen-specific antibodies will naturally decline. As such, while more severe COVID-19 symptoms may elicit longer protection for convalescent individuals, it is plausible that milder symptoms may provide much shorter windows of sterilizing immunity. However, it is established knowledge that adaptive immune responses rely on immunological memory from both B cells and T cells to not only prevent reinfections but also diminish disease severity; this is also the basis of vaccination.

## Antibody-Dependent Enhancement (ADE)

Evidence demonstrating a positive association between high antibody titers and increased clinical severity of COVID-19 has raised the possibility that antibody-dependent enhancement (ADE) could, in some instances, contribute to an excessive immune response that exacerbates SARS-CoV-2 pathogenesis (123, 125, 147–149). ADE is a process in which antibodies bind to viruses to form virus-antibody complexes which potentiates and facilitates host cell entry *via* cell surface Fc receptors, causing infection of Fc-expressing cells such as B cells, dendritic cells, macrophages, and monocytes. Cellular Fc receptors bind to the constant region of antibodies that define the isotype (e.g., Fcγ receptors bind IgG). ADE has been shown to cause increased pathogenicity of some viruses such as Dengue virus, Ebola virus, and Zika virus (150–152). ADE has also been observed in certain human CoV challenges in immunized animals. These include MERS as well as SARS-CoV, where anti-S protein antibodies have potentiated viral entry *via* an ACE2 receptor-dependent mechanism, or independently of ACE2 by facilitating virus uptake *via* FcγRII (153–157). ADE has been observed to induce pro-inflammatory cytokine release from Fc-expressing immune cells in mice and NHPs (157–159). While there is no direct evidence yet to support this hypothesis in the context of COVID-19, the biphasic course of infection that has been described, in which severe hypoxia and respiratory distress typically manifest 7 to 14 days after onset of fever and viremia, coincides with the chronology of seroconversion and IgG class switching (160). Fortunately, animal studies thus far in immunized NHPs re-challenged with SARS-CoV-2 have not shown signs of ADE (149). However, these studies were limited to small numbers of animals and more studies are needed to understand if these animal models can successfully be used to understand ADE in humans. Furthermore, there have been two large-cohort studies published to date on the use of convalescent plasma in human patients, both deeming the incidence of serious adverse events to be low (161, 162). Cumulatively, this would suggest that ADE is unlikely to be a major cause of pathogenesis in SARS-CoV-2 infection in humans. Nevertheless, ADE should be investigated further as it could impact the efficacy and safety of serum therapy, as well as vaccination programs. In particular, if future vaccine candidates are to require booster shots because of impermanent immunity, ADE must be considered as repeated doses generate an increase in antibodies that could potentially contribute to ADE upon virus exposure.

## Cross-Reactivity of SARS-CoV-2 Antibodies to Seasonal CoVs

A significant challenge in developing a specific SARS-CoV-2 serological assay is the potential for cross-reactivity of SARS-CoV-2 capture antigens with antibodies against other human CoVs (163). SARS-CoV-2 shares amino acid sequences and antigenic T and B cell epitopes with the highly prevalent sCoVs that cause the common cold including 229E, OC43, NL63, and HKU1, and also with the now rare MERS and extinct SARS-CoV that both cause severe and fatal respiratory disease (**Figure 2**) (62, 164–168). A recent prevalence survey of the sCoVs using RT-PCR revealed that OC43 is the most prevalent sCoV followed by NL63, HKU1, and finally 229E (169). While infection with the sCoVs induce antibody responses as would be expected, these wane over time and render the hosts susceptible to reinfection. An impressive study of the occurrence of reinfection for all four sCoVs over more than a 35 year span revealed that reinfections with the same sCoV occurred most frequently after 12 months (12). While sterilizing immunity to sCoVs is relatively short-lived, here we will review current knowledge about cross-reactivity of these antibodies to SARS-CoV-2 antigens.

Given that the prevalence of antibodies against all four sCoVs may be as high as 90%, as demonstrated in one sample of American adults (170), antigens used in SARS-CoV-2 serological assays may in some instances be detected by these naturally circulating and highly prevalent antibodies, thereby limiting test specificity and creating the potential for false-positive results. While the greatest probability for cross-reactivity exists between antibodies directed against SARS-CoV-2, SARS-CoV, and MERS, the latter two are exceedingly rare given the low case numbers of these infections (171, 172). Therefore, issues related to cross-reactivity between SARS-CoV-2 and the circulating sCoVs are of foremost concern.

Several serological studies have demonstrated cross-reactivity of SARS-CoV-2 S protein with SARS-CoV, MERS, and sCoVs (43, 53, 116, 118, 165, 173–175). The spike S2 domain is believed to be primarily responsible for this cross-recognition given its slightly higher level of sequence similarity than the other S domains (118). When comparing the amino acid sequence by both percent identity and percent similarity of all human CoVs, the S2 domain has the highest identity and similarity compared to full S, S1, RBD, or N domains (**Tables 1** and **2**) (173). Cross-reactivity between SARS-CoV-2 antibodies and the S2 domain of the SARS-CoV S protein has also been shown (176). Some studies examined the specificity of ELISAs and demonstrated cross-reactivity of sCoVs, MERS, and SARS-CoV sera only with the full SARS-CoV-2 S protein, and not with the S1 antigen (53, 173), which is in agreement with several other groups that failed to measure cross-reactivity of sCoV antibodies with SARS-CoV-2 RBD (62, 134, 176). Importantly, the lack of cross-reactivity demonstrated between the S1 subdomain of SARS-CoV-2 and sCoVs antibodies may point to its potential application as a target antigen for highly specific serological assays.

**Table 1:** Percent identity of amino acid sequences between human CoVs and SARS-CoV-2.

	Alphacoronavirus		Betacoronavirus			
	229E	NL63	OC43	HKU1	MERS	SARS
**Full spike**	31.4	29.8	30.2	29.5	34.8	76.0
**S1 Domain**	31.2	25.0	23.8	23.7	28.3	60.3
**S2 Domain**	35.0	33.1	42.3	41.2	43.6	90.0
**RBD**	24.1	27.8	23.8	29.4	21.7	73.1
**Nucleoprotein**	28.4	32.6	34.6	33.9	49.7	90.5

Alignments between amino acid sequences of all 7 human coronavirus were done for the Full spike, spike domains S1, S2, and RBD and the nucleoprotein (N).

**Table 2:** Percent similarity of amino acid sequences between human CoVs and SARS-CoV-2.

	Alphacoronavirus		Betacoronavirus			
	229E	NL63	OC43	HKU1	MERS	SARS
**Full spike**	61.8	60.0	57.9	58.0	65.7	91.5
**S1 Domain**	62.5	51.4	51.7	55.6	61.9	84.2
**S2 Domain**	66.5	66.3	72.7	72.7	73.9	98.1
**RBD**	59.3	59.3	54.7	67.6	56.0	88.9
**Nucleoprotein**	57.9	65.1	62.1	65.1	75.4	97.2

Alignments between amino acid sequences of all seven human coronavirus were done for the Full spike, spike domains S1, S2, and RBD and the nucleoprotein (N).

There may also be cross-reactivity issues that affect the specificity of tests that use the N protein. The N protein of SARS-CoV-2 shows 97% similarity to that of SARS-CoV, 75% to MERS, and 58% to 65% similarity to the sCoVs (**Table 2**). One previous study analyzed the cross-reactivity of the N protein between the various CoV groups (177). They found that antibodies against the seasonal alphacoronaviruses 229E and NL63 demonstrated cross-reactivity towards each other but did not cross-react with betacoronavirus antigens, which would include SARS-CoV-2. Meanwhile, betacoronavirus (NL63 and OC43) sera primarily cross-reacted with N proteins from other betacoronaviruses, with the exception of SARS-CoV (177). While the SARS-CoV-2 N antigen was not included in the study, this suggests that it is likely that other betacoronaviruses would similarly cross-react with SARS-CoV-2. Indeed, *Ng et al.* also showed that sCoV-reactive serum could bind to the SARS-CoV-2 N protein in their flow cytometry-based detection assay (20). Therefore, there is also the potential for cross-reactivity between pre-existing antibodies towards sCoVs and the N antigen of SARS-CoV-2. While the high abundance of the N protein otherwise makes it a promising candidate for diagnostic serological assays, the potential for poor specificity due to cross-reactivity with prevalent sCoVs may be a critical limitation to its use. Indeed, in a pre-print manuscript, Anderson *et al.* demonstrated in a cohort of 207 pre-pandemic samples that 5% reacted to the SARS-CoV-2 S proteins, 2% against the RBD, and 19% against N (116). A closer analysis of these samples revealed that most had antibodies against OC43, 229E, and NL63 but these were non-neutralizing.

## Protection From SARS-CoV-2 Infections by sCoVs Antibodies

While there is strong evidence that antibodies raised against sCoVs antigens can bind to SARS-CoV-2 proteins and interfere with serological assays, there is conflicting information concerning the protective role of these sCoV antibodies. While most neutralizing antibodies target the RBD to disrupt binding to the host-expressed ACE2 receptor (61, 178, 179), cross-reactive sCoVs primarily target the S2 domain of the SARS-CoV-2 spike protein (118, 167). Furthermore, non-RBD S1 as well as S2-binding neutralizing antibodies have been identified for both SARS-CoV and SARS-CoV-2 (92, 165, 180–182). As of this moment, two studies have shown evidence that the presence of sCoV antibodies is associated with less severe COVID-19 symptoms (183, 184), while two more have shown some neutralizing activity in pre-pandemic samples (118, 165).

More specifically, Ng *et al.* showed that healthy individuals with recent sCoV exposure had antibodies capable of limiting SARS-CoV-2 entry into host cells in an experimental system (118). It has been proposed that the protection conferred by sCoV antibodies may also contribute to the age disparity in COVID-19 susceptibility (118, 185). Seroprevalence of sCoVs varies considerably between age groups, with especially high prevalence in very young children (<1 year of age), an observation that aligns well with the fewer number of severe cases of COVID-19 in children (186, 187). The high prevalence of protective sCoV antibodies in younger individuals provides a plausible explanation for why young adults under the age of 20 are estimated to be half as susceptible to COVID-19 as those above the age of 20, and why children comprise only 21% of symptomatic cases, compared to upwards of 69% for those above 70 years of age (188). This hypothesis, however, remains to be tested in a well-designed randomized clinical trial.

However, mounting evidence support that few to no cross-neutralizing sCoV antibodies do in fact exist (116, 117). Nevertheless, one must be cognizant that most neutralizing assays utilize spike-pseudotyped viruses or surrogate (virus-free) assays with purified antigen. It is possible that such experimental systems fail to measure the overall protection of sCoV pre-exposure as seen in a living person. Indeed, sCoV-induced cross-reactive T cell responses and Fc effector functions of antibodies may also play a role in COVID-19 severity and outcomes (62, 164, 166, 168).

## Immunological Back-Boosting by sCoV Antibodies and the Original Antigenic Sin

As described above, cross-reactivity of sCoVs antibodies to SARS-CoV-2 antigens has now been well characterized. It is also well established that most people have had prior exposure and produce antibodies to several sCoVs. While there is some evidence that sCoV antibodies can neutralize SARS-CoV-2, so far neutralization appears to be weak if at all detectable. Given that most cross-reactive antibodies bind the S2 region of the SARS-CoV-2 spike and are non-neutralizing, a very interesting an important question arises: can this cross-recognition of antigens give rise to immunological imprinting?

Immunological imprinting, also called original antigenic sin, relates to the concept of mounting an antibody response to a new pathogen using memory cells recognizing past antigens over stimulating a *de novo* antibody response (189). Such responses have been shown for influenza and dengue virus and are associated with poor virus neutralization and can have profound consequences on vaccine efficacy (190–193). For SARS-CoV-2 infections, a number of studies have now reported back-boosting of non-neutralizing sCoV antibody production (116, 118, 167, 194, 195). These antibodies appear to be most prominently targeted against conserved epitopes in OC43 and HKU1, both betacoronaviruses (194, 195). Interestingly, none of these studies presented evidence that a sCoV antibody boost was associated with either protection against SARS-CoV-2 infection or COVID-19 severity. In fact, a negative correlation was observed in some studies, providing additional support to a disfavorable consequence of immunological imprinting (194, 195). Given that most of the leading SARS-CoV-2 vaccine candidates being currently developed use the full (S1-S2) spike protein as the primary viral antigen, special consideration needs to be given as to whether inclusion of the S2 domain will be a factor that that impedes vaccine efficacy.

## Epidemiological Implications of Serology Testing for SARS-CoV-2

As the COVID-19 pandemic continues around the world, with second and third waves of infections already taking form, it is becoming increasingly important to monitor serological data at the population level. Effective and ethical response strategies to the COVID-19 pandemic can only be formulated once it is accurately determined if neutralizing antibodies are present, how effective those antibodies are at preventing disease and viral spread, and how long that immunity will last. Ongoing epidemiological considerations include the concepts of herd immunity, shield immunity, and immunity passports. However, these ideas remain largely based on the assumption that a humoral response implies lasting immunity, making their implementation for SARS-CoV-2 premature on an ethical basis. It must be reiterated that further functional serological studies must be performed to measure long-term effectiveness of humoral responses.

One of the most widely discussed epidemiological concepts surrounding COVID-19 is the possibility of achieving herd immunity. Herd immunity is a population-level phenomenon where the risk of infection for susceptible and disproportionately vulnerable individuals is mitigated by the presence and proximity of immune individuals. As more people develop immunity, the risk to susceptible population decreases, resulting in fewer opportunities for pathogen transmission (196). Herd immunity is particularly important for protecting those who cannot be effectively vaccinated, such as the very young and the immunocompromised (197). For COVID-19, where the majority of deaths and severe symptoms are observed in patients 60 and older (198), herd immunity will also play an important role in protecting the vulnerable elderly population.

In order to achieve protection, a minimum percentage of the population, known as the herd immunity threshold (HIT), must develop immunity. In its most basic form, the HIT is estimated with the formula (R_0_ – 1)/R_0_, where R_0_ (the basic reproduction number for an infectious disease) represents the number of secondary cases generated by each infected individual in a fully susceptible population (199). Early models investigating the localized outbreaks in China estimated R_0_ for COVID-19 to range from 1.4 to 6.49, with a mean value of 3.28 (threshold = 69.5%) (200). While estimates continue to vary, there is a general consensus that the average value of R_0_ for COVID-19 is approximately between 2 and 3, implying that a minimum of 50% to 67% of a population must achieve immune resistance before herd immunity can take effect (201).

Herd immunity can either be achieved through natural acquisition (i.e., natural herd immunity) or by controlled vaccination programs (202). Natural herd immunity assumes that convalescence imparts sterilizing immunity, and therefore widespread infection is necessary for widespread immunity. In the context of COVID-19, while natural herd immunity is theoretically possible, its pursuit is difficult to ethically justify given the high mortality and lasting morbidity caused by the virus, and it is also difficult to implement practically. Recent seroprevalence data shows that no country is even close to achieving herd immunity through natural acquisition (**Table 3**). In Sweden, where no official lockdown measures were enforced throughout the pandemic, as of May 2020, there was only a seroprevalence of 15% in Stockholm (compared to their predicted seroprevalence of 40%) (214). Furthermore, in several COVID-19 “hotspots” like Iran and New York City, where large numbers of cases were observed in short timespans, seroprevalence still never exceeded 25%. In the vast majority of other cities and countries, seroprevalence is usually much lower than 10% (**Table 3**). Most importantly, given the high risk of long-term morbidity due to tissue damage caused by COVID-19, naturally acquired herd immunity cannot be ethically pursued or encouraged (215–218). Therefore, the pursuit of natural herd immunity against SARS-CoV-2 is not justifiable in any form or manner and will be associated with very high immediate and long-term healthcare costs due to chronic disease.

**Table 3:** Seroprevalence of antibodies against SARS-CoV-2 in various countries and cities.

Location	Seroprevalence (%)^1^	N^2^	Reference
**Iran **	22.89	5877	(203)
**Switzerland** ** Geneva**	7.58 10.8	16758 775	(203) (204)
**United Kingdom** ** London**	6.94 13	182072 9547	(203) (205)
**Sweden** ** Stockholm**	5.6 11.5	1200 >100^3^	(206)
**USA** ** New York City**	4.89 19.5	4.89 1581	(203) (207)
**Spain** ** Madrid**	4.66 11.3	1018251 3186	(203) (208)
**France**	2.78	5534	(203)
**Netherlands**	2.77	36791	(203)
**Brazil**	1.29	157360	(203)
**Canada** ** Montreal** ** Toronto** ** Vancouver**	1.06 3.05 1.5 0.55	50269 7691 1837 885	(203) (209) (210) (211)
**Italy** ** Milan**	1.04 5.2	904 789	(203) (212)
**China** ** Wuhan**	0.8 2.29	10449 17794	(203) (213)
**Iceland** ** Reykjavik**	0.3 0.4	18609 4843	(135)

A sampling of published SARS-CoV-2 seroprevalence studies are listed below to demonstrate the wide range of values observed around the world and relatively low worldwide seropositivity.

^1^Reported seroprevalences reflect the most recent data available, although some values are from as early as April 2020.

^2^Sample sizes for data from **SeroTracker** (203) are summations of the “N” column values for all relevant studies included at the time of publication;

^3^The exact sample size was not reported, simply that it was greater than 100.

A safer, more effective, and ethically sound alternative to acquiring natural herd immunity is to deploy controlled vaccination programs. Rigorously tested and formally approved vaccines offer a safe and effective method to quickly increase a population’s immunity to a harmful pathogen (219). Furthermore, vaccines are designed to elicit a neutralizing antibody response without the severe pathogenesis associated with the corresponding disease, in this case, COVID-19. Therefore, while it may take several more months for a safe and effective SARS-CoV-2 vaccine to be developed, tested and deployed, it is widely agreed upon that vaccine-acquired herd immunity is faster, safer, cheaper, and more effective than natural herd immunity. However, cautious optimism is warranted over vaccines. Based on knowledge acquired from SARS-CoV convalescent individuals, long-term protective immunity may last for only a few months (6, 9). With waning humoral immunity over time against a highly prevalent and infectious virus, maintaining herd immunity at the population level will almost certainly require booster shots and updated vaccines to maintain immunity against reinfections by SARS-CoV-2 and its inevitable genetic variants that are poised to emerge.

## Conclusions

Significant progress has been made with respect to understanding the antibody-mediated immune response to SARS-CoV-2 infections. The applications and utility of serological assays are manifold, spanning from the development of screening modalities for epidemiological monitoring and drafting effective public health policy, to the creation of vaccines, and finally to the diagnosis of past infections.

However, the appropriate utilization of serological data requires an understanding of its limitations and ensuring these limitations are accounted for in the current and future pandemic response. For example, it remains unclear whether differences exist between the effectiveness and duration of immunity procured by a natural SARS-CoV-2 infection or vaccine-mediated immunity. Furthermore, existing serological testing approaches are widely varied in their sensitivity, specificity, and practicality, and require an adept understanding of the characteristics of each test in order to determine which should be suitably used in which context. Finally, studying the characteristics of the main SARS-CoV-2 antigens has revealed how some may be better suited for either vaccine development versus serological testing. However, cross-reactivity to sCoVs, the risk of ADE, and emergence of mutations will have profound implications on how these antigens should be employed in vaccination or screening technologies.

As the COVID-19 pandemic continues to impart substantial human suffering and economic losses throughout the world, various governments and stakeholders are experiencing increasing urgency and pressure to re-open commercial and social activities. Nevertheless, folding under this pressure has the risk of driving pre-emptive action based on inconclusive evidence, as large-scale policy mistakes such as encouraging natural herd immunity, the implementation of unstandardized serological assays, or the distribution of unproven immunity passports may reverse progress and incur unacceptable human and financial costs. Instead, this motivation to resolve the pandemic should prompt the thoughtful application of existing research, as well as support initiatives that seek to address the remaining evidence gaps within epidemiology and questions of long-term immunity to COVID-19. Such a grounded approach will be required if we are to create safe and effective solutions for the rapid diagnosis and prevention of COVID-19 and, ultimately, return to our daily activities without having to wear a mask.

## Author Contributions

YG and MG equally carried out the literature review, prepared the figures and tables, and the drafting of the manuscript. GL and MD contributed to the drafting of the manuscript. M-AL contributed to the literature review and drafting of the manuscript. All authors contributed to the article and approved the submitted version.

## Conflict of Interest

The authors declare that the research was conducted in the absence of any commercial or financial relationships that could be construed as a potential conflict of interest.
