# Supplementary material for: Humoral Responses and Serological Assays in SARS-CoV-2 Infections
Source: Front Immunol. 2020 Dec 18;11:610688. doi: 10.3389/fimmu.2020.610688 (PMC7775512; doi:10.3389/fimmu.2020.610688)
Supplement: Supplementary file 1 [file Table_1.pdf]

Humoral Responses and Serological Assays in SARS-CoV-2 Infections

Yannick Galipeau[1,*], Matthew Greig[1,*], George Liu[1], Matt Driedger[2] and Marc-André Langlois[1,3]

SUPLEMENTARY MATERIAL

Table S1: Approved Tests For SARS-CoV-2 Serology

Company	Test Name	Approvals	Test Type	Antigen	Ig	Specificity	Sensitivity	PPV/PPA	NPV/NPA	URL
Abbott	Alinity i SARS-CoV-2 IgG	USA, Canada, UK, EU, Australia	CLIA	N	IgG	99%	100%	84%	100%	https://www.corelaboratory.abbott/us/en/offerings/segments/infectious-disease/sars-cov-2
Abbott	Architect SARS-CoV-2 IgG	USA, Canada, UK, EU, Australia	CLIA	N	IgG	99.6%	100%	92.9%	100%	https://www.corelaboratory.abbott/us/en/offerings/segments/infectious-disease/sars-cov-2
Abingdon Health	AbC-19 Rapid Test	UK, EU	LFA	Sp	IgG	99.56%	98.03%			https://www.abingdonhealth.com/uk-covid-19-rapid-antibody-tests-approved-for-professional-use/
ADS Biotec	SARS-CoV-2 IgM/IgG Antibody Test Kit	USA, EU	LFA	Viral Antigen	IgG and IgM	99.5%	97.5%			http://www.adsbiotec.com/products/sars-cov-2-test-kits/
Assure Tech	Assure COVID-19 IgG/IgM	USA	LFA	N +S1	IgG and IgM	IgG 100%<br>IgM 98.8%	IgG 90%<br>IgM 100%	80.8%	100%	https://www.fda.gov/media/139792/download
Autobio Diagnostics	Anti-SARS-CoV-2 Rapid Test	EU	LFA	Sp	IgG and IgM	IgG 99.4%<br>IgM 99.7%	IgG 99%<br>IgM 95.7%	84.4%	99.9%	http://www.autobio.com.cn/en/
Babson Diagnostics	Babson Diagnostics aC19G1	USA	CLIA	?	IgG	100%	100%	100%	100%	https://www.fda.gov/media/139446/download
Beckman Coulter	Access SARS-CoV-2 IgG	USA	CLIA	RBD	IgG	99.6%	96.8%	92.7%	99.2%	https://www.fda.gov/media/139627/download
Beijing Wantai	Wantai SARS-CoV-2 Ab Rapid Test	USA	LFA	Viral Antigen	Pan-Ig	98.8%	100%	80.8%	100%	https://www.fda.gov/media/140030/download
Beijing Wantai	Wantai SARS-CoV-2 Ab ELISA	USA	ELISA	RBD	Pan-Ig	97.5%	96.7%	67.1%	99.8%	https://www.fda.gov/media/140929/download
Beroni Group	SARS-CoV-2 IgG/IgM Antibody Detection Kit	EU	LFA	?	IgG and IgM	88.57%	100%			https://www.beronigroup.com/covid-19/
BioCheck Inc	BioCheck SARS-CoV-2 IgG IgM Combo Test	USA, EU	CLIA	S1	IgG and IgM	IgG 100%<br>IgM 97.2%	IgG 100%<br>IgM 89%	IgG 100%<br>IgM 89%	IgG 97.2%<br>IgM 100%	https://www.fda.gov/media/141251/download
Biohit Healthcare	Biohit SARS-CoV-2 IgM/IgG Test Kit	USA	LFA	N	IgG and IgM	95%	96.7%	50.4%	99.8%	https://www.fda.gov/media/139283/download
Biolidics	2019-nCoV IgG/IgMDetection Kit (Colloidal Gold)	EU	LFA	Viral Antigen	IgG and IgM	97%	91.5%			https://www.biolidics.com/2019-ncov-igg-igm-antibody-detection-kit

BioMedomics	COVID-19 IgM-IgG Rapid Test	EU	LFA	Viral Antigen	IgG and IgM	97%	96.7%			https://www.biomedomics.com/products/infectious-disease/covid-19-rt/
Biomerica	COVID-19 IgG/IgM Rapid Test	EU	LFA	?	IgG and IgM	100%	87%			http://www.biomerica.com/products/product_detail.asp?ProductID=154
BioMerieux	Vidas anti-SARS-CoV-2 IgG Test	USA, EU	ELFA	?	IgG	99.9%	100%	98.1%	100%	https://www.biomerieux-diagnostics.com/vidas-sars-cov-2
Biomerieux	Vidas anti-SARS-CoV-2 IgM Test	USA, EU	ELFA	?	IgM	99.4%	100%	89%	100%	https://www.biomerieux-diagnostics.com/vidas-sars-cov-2
BioPanda Reagents	COVID-19 Rapid Antibody Test	EU	LFA	Viral Antigen	IgG and IgM	IgG 98.3% IgM 96.7%	100%			https://www.biopanda.co.uk/php/products/rapid/infectious_diseases/covid19.php
Bio-Rad Labs	Platelia SARS-CoV-2 Total Ab	USA, Australia	ELISA	N	Pan-Ig	99.6%	92.2%	88.6%	99.9%	https://www.bio-rad.com/featured/en/sars-cov-2-surveillance.html
Cellex	qSARS-CoV-2 IgG/IgM Rapid Test	USA, Australia,	LFA	Sp + N	IgG and IgM	95.6%	93.8%	91.7%	99.6%	https://cellexcovid.com/product/
CTK Biotech	OnSite COVID-19 IgG/IgM Rapid Test	EU, Australia	LFA	Viral Antigen	IgG and IgM	IgG 100% IgM 100%	IgG 98.8% IgM 88.2%	Combo 46.8%	Combo 99.6%	https://ctkbiotech.com/product/onsite-covid-19-igg-igm-rapid-test/
DiaSorin Inc	Liaison SARS-CoV-2 S1/S2 IgG	USA, Canada, EU	CLIA	Sp	IgG	99.3%	97.6%	87.5%	99.9%	https://www.diasorin.com/en/immunodiagnostic-solutions/clinical-areas/infectious-diseases/covid-19
Diazyme Laboratories	Diazyme DZ-Lite SARS-CoV-2 IgG	USA	CLIA	Viral Antigen	IgG	97.4%	100%	67.1%	100%	https://www.fda.gov/media/139865/download
Emory Medical	SARS-CoV-2 RBD IgG Test	USA	ELISA	RBD	IgG	96.4%	100%	59.3%	100%	https://www.fda.gov/media/139053/download
Erba Mannheim	ErbaLisa COVID-19 ELISA Kits	EU	ELISA	N	IgG and IgM	98%	98%	88%	99.9%	https://erbalisacovid19.erbamannheim.com/
Eurofins Inganasa	Ingezim COVID 19 DR	EU	ELISA + LFA	N	Pan Ig	99.2%	98.3%			https://www.eurofins-technologies.com/ingezim-covid-19-dr.html
EUROIMMUN	SARS-CoV-2 ELISA (IgG)	USA, EU, Australia	ELISA	S1	IgG	100%	90%	100%	99.5%	https://www.euroimmun.com/products/infection-diagnostics/pd/emerging-diseases/2606/2/69492/
GenScript Biotech	cPass SARS-CoV-2 Neutralization Antibody Detection Kit	EU	ELISA	S1 RBD	Total Neutralizing Ig	94%	96.7%	100%	99.5%	https://www.genscript.com/covid-19-detection-svnt.html

Gold Standard Diagnostics/ Eurofins Technology	NovaLisa SARS-CoV-2 IgG, IgA, IgM Assays	EU	ELISA	Viral Antigens	IgG, IgM or IgA	IgG >98%	IgG 100%			https://www.eurofinsgenomics.eu/en/gene-synthesis-molecular-biology/applications/sars-cov-2-elisa-kits/
Hangzhou AllTest Biotech	Rightsign 2019-nCoV IgG/IgM Rapid Test Cassette	USA, EU, Australia	LFA	Sp	IgG and IgM	IgG 98%<br>IgM 96%	IgG 100%<br>IgM 85%	100%	100%	http://en.biotests.com.cn/
Healgen Scientific LLC	COVID-19 Antibody Rapid Detection Kit	USA, Australia	LFA	Sp	IgG and IgM	97.5%	100%	100%	100%	https://www.healgen.com/if-respiratory-covid-19
InBios International	SCoV-2 Detect IgG/IgM Tests	USA	ELISA	Viral Antigen	IgG or IgM	IgG 100%<br>IgM 98.8%	IgG 100%<br>IgM 96.7%	IgG 97.8%<br>IgM 92.5%	IgG 98.9%<br>IgM 98.9%	https://www.fda.gov/media/138810/download<br>https://www.fda.gov/media/139730/download
Kabla Clinical Diagnostics	Rapid Test for Coronavirus IgG IgM Antibodies	EU,	LFA	Viral Antigen	IgG and IgM	IgG 98%<br>IgM 96%	IgG 99.9%<br>IgM 85%			https://kabla.mx/covid-test/
Luminex	xMAP SARS-CoV-2 Multi-Antigen IgG	USA	MIA	N, RBD and S1	IgG	99.2%	100%	88.4%	99.8%	https://www.fda.gov/media/140256/download
Mediagnost	Anti-SARS CoV-2 ELISA	EU	ELISA	S1 RBD	IgG	98.6%	95.3%	67.8%	100%	https://mediagnost.de/en/anti-sars-cov-2-elisa/
MedMira	REVEALCOVID-19 Total Antibody Test	EU	VFA	Viral Antigen	Pan Ig	98.8%				https://revealcovid19.com/
Megna Health Inc.	Rapid COVID-19 IgM/IgG Combo Test Kit	USA	LFA	N	IgG and IgM	IgG 97.5%<br>IgM 97.5%	IgG 100%<br>IgM 83.3%	51.3%	100%	https://www.fda.gov/media/140297/download
Mount Sinai Hospital Clinical Laboratory	Mt. Sinai Lab COVID-19 ELISA Antibody Test	USA	ELISA	Sp	Pan Ig	100%	92.5%	97%	98%	https://www.mountsinai.org/about/covid19/antibody-test
Nad von minden	Nadal COVID-19 IgG/IgM test	EU	LFA	Viral Antigen	IgG and IgM	99.2%	94.1%	100%	99.6%	http://www.nal-vonminden.com/pdf/EN-COVID19-Infoflyer.pdf
Ortho-Clinical Diagnostics Inc	VITROS Anti-SARS-CoV-2 IgG Test	USA, EU	CLIA	Sp	IgG	100%	90%	100%	99.5%	https://www.orthoclinicaldiagnostics.com/global/covid19/antibody-test
Ortho-Clinical Diagnostics Inc	VITROS Anti-SARS-CoV-2 Total Test	USA, Canada, EU	CLIA	Sp	Pan-Ig	100%	100%	100%	99.5%	https://www.orthoclinicaldiagnostics.com/global/covid19/antibody-test
PerkinElmer	SuperFlex™ Anti-SARS-CoV-2 IgG Kit	EU	CLIA	Viral Antigen	IgG	100%	100%	90.3%		https://perkinelmer-appliedgenomics.com/home/products/superflex-immunoassay-system-ce-ivd/superflex-sars-cov-2-antibody-

										detection-kits-ce-ivd/
PerkinElmer	PerkinElmer GSP/DELFIA Anti-SARS-CoV-2 IgG Kit	EU	DBS ELFA	Viral Antigen	IgG	98.7%	96.2%			https://perkinelmer-appliedgenomics.com/home/sars-cov-2-testing-solutions/perkinelmer-gsp-delfia-anti-sars-cov-2-igg-kit/
Quotient	Mosaiq COVID-19 Antibody Microarray	EU	MIRA	?	IgG and IgM	99.8%	100%	100%	100%	https://quotientbd.com/covid-19
Roche	Elecsys Anti-SARS-CoV-2	USA, Canada, UK,	ECLIA	N	Pan-Ig	99.8%	100%	96.5%	100%	https://diagnostics.roche.com/global/en/products/params/elecsys-anti-sars-cov-2.html
Salofa Oy	Sienna-Clarity COVIBLOCK COVID-19 IgG IgM Rapid Test	USA	LFA	RBD	IgG and IgM	IgG 98.8% IgM 100%	IgG 93.3% IgM 90%	79.7%	99.6%	https://www.fda.gov/media/140082/download
Siemens Healthcare Diagnostic Inc	Attelica IM / ADVIA Centaur SARS-CoV-2 Total Assay	USA, Canada, EU	CLIA	S1 RBD	IgG and IgM	99.8%	100%	96.5%	100%	https://www.siemens-healthineers.com/en-ca/laboratory-diagnostics/assays-by-diseases-conditions/infectious-disease-assays/cov2t-assay
Snibe Diagnostics	Maglumi 2019 nCoV (SARS-CoV-2) IgM/IgG Kits	EU	CLIA	?	IgG and IgM	96%	89-95%	96.6%	100%	http://www.snibe.com/zh_en/en_newsView.aspx?id=576
SureScreen Diagnostics	SureScreen COVID19 IgM/IgG Rapid Test Cassette	EU	LFA	Viral Antigen	IgG and IgM	99%	91%			https://www.surescreen.com/products/covid-19-coronavirus-rapid-test-cassette
Vibrant America	Vibrant COVID-19 Antibody Test	USA	CLIA	S1, RBD, S2, and N	IgG and IgM	98.6%	98.1%	78.7%	99.9%	https://www.vibrant-america.com/covid-19-antibody/
Wadsworth Center, NY State Dep. of Health	NY Sars-CoV Microsphere Immunoassay for Antibody Detection	USA	MIA	N	Pan-Ig	98.8%	88%	79.4%	99.4%	http://dmna.ny.gov/covid19/docs/all/DOH_COVID19_AntibodyTestingFactSheet_041420.pdf
Xiamen Biotime Biotech	Biotime SARS-CoV-2 IgG/IgM Rapid Qualitative Test	USA	LFA	Viral Antigen	IgG and IgM	96.2%	100%	58.4%	100%	https://www.fda.gov/media/140443/download

Abbreviations:

DBS: Dried Blood Spot
ECLIA: Electrochemiluminescence Immunoassay
ELFA: Enzyme Linked Fluorescent Assay
ELISA: Enzyme Linked Immunosorbent Assay
ELFA: Enzyme Linked Fluorescent Assay
LFA: Lateral Flow Assay
MIA: Microsphere Immunoassay
MIRA: Multiplexed Immuno-Refractory Assay
NPV/NPA: Negative Predictive Value
PPV/PPA: Positive Predictive Value
VFA: Vertical Flow Assay

USA = FDA EUA Granted
EU = CE-IVD Granted
Can = Health Canada Approved
UK= England Public Health Approved
Australia = Australia Public Health Approved
